# High throughput, small scale methods to characterise the growth of marine fungi

**DOI:** 10.1371/journal.pone.0236822

**Published:** 2020-08-07

**Authors:** Anu Tamminen, Petrus Happonen, Dorothee Barth, Sami Holmström, Marilyn G. Wiebe

**Affiliations:** VTT Technical Research Centre of Finland Ltd, Espoo, Finland; Leibniz-Institut fur Naturstoff-Forschung und Infektionsbiologie eV Hans-Knoll-Institut, GERMANY

## Abstract

Various marine fungi have been shown to produce interesting, bioactive compounds, but scaling up the production of these compounds can be challenging, particularly because little is generally known about how the producing organisms grow. Here we assessed the suitability of using 100-well BioScreen plates or 96-well plates incubated in a robot hotel to cultivate eight filamentous marine fungi, six sporulating and two non-sporulating, to obtain data on growth and substrate (glucose, xylose, galactose or glycerol) utilisation in a high throughput manner. All eight fungi grew in both cultivation systems, but growth was more variable and with more noise in the data in the Cytomat plate hotel than in the BioScreen. Specific growth rates between 0.01 (no added substrate) and 0.07 h^-1^ were measured for strains growing in the BioScreen and between 0.01 and 0.27 h^-1^ for strains in the plate hotel. Three strains, *Dendryphiella salina* LF304, *Penicillium chrysogenum* KF657 and *Penicillium pinophilum* LF458, consistently had higher specific growth rates on glucose and xylose in the plate hotel than in the BioScreen, but otherwise results were similar in the two systems. However, because of the noise in data from the plate hotel, the data obtained from it could only be used to distinguish between substrates which did or did not support growth, whereas data from BioScreen also provided information on substrate preference. Glucose was the preferred substrate for all strains, followed by xylose and galactose. Five strains also grew on glycerol. Therefore it was important to minimise the amount of glycerol introduced with the inoculum to avoid misinterpreting the results for growth on poor substrates. We concluded that both systems could provide physiological data with filamentous fungi, provided sufficient replicates are included in the measurements.

## Introduction

The marine environment is a source of organisms which produce novel bioactive compounds and interesting proteins [1, 2]. Much attention has been focused on marine bacteria, but interest in marine fungi has grown in recent years as isolation and collection of these has increased [3–5]. These fungi are diverse and many isolates may be opportunistic, rather than obligate marine organisms [5–8]. None-the-less, several unique bio-active compounds [7, 9, 10] and enzymes with unique properties [11–13] have been isolated from marine isolates, which had not been found in their terrestrial cousins.

Screening for novel compounds often involves a large selection of organisms cultivated in a limited range of conditions or a few organisms cultivated in a wider variety of conditions including stress; it may also include genome sequencing and analysis [14–16]. The focus of biodiscovery is on the metabolites or proteins of interest, rather than on the producing organism. As a result, when an interesting compound is identified and a large amount of the compound is needed to enable further testing or development, there may be no information about the growth of the strain, beyond the conditions used in the initial screening [9]. These screening conditions may be unsuitable for large scale production [17, 18]. Information about specific growth rate on specific substrates and the extent to which the substrate is utilised would be helpful in the development of scalable production conditions. This is particularly the case with products which are associated with the cell biomass, rather than secreted into the culture supernatant, since good biomass production may be a requirement for achieving high levels of product [19, 20]. Good growth does not guarantee high product amounts, but is necessary to provide sufficient bio-catalyst to initiate production.

Obtaining data on growth kinetics for unicellular yeast and bacteria has been greatly facilitated by the development of microtiter plates and associated tools for automation. Growth in microtiter plates is generally measured as optical density. Microtiter plates have less frequently been used to obtain kinetic data for filamentous fungi, which may not grow uniformly in the wells. Filamentous fungi grow as branched mycelia, without cell separation at division. In liquid, mycelia may remain dispersed or form pellets [21, 22]. In addition, fungal hyphae are adept at attaching to surfaces; in a small volume, such as in a 96-well microtiter plate, the ratio of attached biomass to submerged biomass may be high. If agitation is inadequate, filamentous fungi may also grow as mats on the surface of the liquid. Heterogeneity is inherent to all forms of mycelial growth and heterogeneity impacts the suitability of optical density as a tool for measurement of growth, which is sensitive to particle size [23]. None-the-less, Trinci and colleagues [24–28] demonstrated that optical density provides a meaningful measure of fungal growth for strains growing as dispersed mycelia. Banerjee et al. [29] expanded the use of optical density to monitor the growth of filamentous fungi by adding a homogenisation step to the measurement, noting that the extent of fragmentation would impact the relationship between optical density and cell dry biomass. Fuentes et al [30] found optical density measurements more practical than microscopic methods when assessing the growth of a number of marine fungi.

Microtiter plates are used with filamentous fungi as well as with unicellular microbes, but their use has often been limited to isolation [31] or monitoring plus/minus growth (e.g. [32]), rather than providing kinetic data which could be used to scale up a process. The 24-well plate is starting to replace flasks for fungal cultivation and is reported to be equivalent to or better than flasks for generating reproducible data on intracellular [33, 34] and extracellular [35–38] metabolites and proteins [39, 40]. 24-well plates typically require removal from the incubator to obtain measurements of growth, e.g. once or twice per day, but may be used to assess products only, without providing information on biomass production and growth. The BioLector 48-well plate, which provides growth information *in situ*, has also been used effectively with the filamentous fungi *Aspergillus terreus* [41] and *Penicillium chrysogenum* [42], but is limited to single plate incubation.

The 96-well format has been used to study fungal biofilms [43, 44], fungicide sensitivity [44–50], metabolite production [51–53] enzyme secretion [54] and comparison of strains based on sporadic OD measurements [50, 55]. More detailed growth curves have also been obtained [56]. In addition, BioLog 96-well plates, which provide a range of predefined substrates (carbohydrates, nitrogen sources, potential inhibitors, depending on the plate selected), have been used to characterise or compare various fungi including *Trichoderma* spp. [57, 58], *Oidiodendron fimicola* [59], *Beauveria brongniartii* [60], *Petriella setifera* [61], and environmental isolates [62–66]. The BioLog plates have provided some information on growth characteristics, but have primarily been used to assess the metabolic potential of strains and to distinguish between isolates. Some marine fungi have been characterised using BioLog FF (filamentous fungi) plates [30, 67].

While 96-well plates are primarily used for basic screening (product, substrate utilisation, tolerance, etc.), the BioScreen 100-well honeycomb plates are used to assess growth characteristics [68–71], as well as for screening [72, 73]. BioScreen readers hold only two plates, restricting simultaneous measurement to 200 wells. This is an improvement compared to the 48-well BioLector plate, but restrictive compared to the potential of most 96-well plate incubators.

Some fungi grow filamentously in the 96 and 100-well plates, but aids may be added to improve the nature of growth and quality of the growth curve; these include Tween 80 [56] and agar [68], with the recommendation that appropriate conditions be determined for each strain to be evaluated [68].

Here we present a comparison of the use of 100-well BioScreen plates and 96-well plates incubated in a Cytomat plate hotel to assess the growth of several filamentous fungi isolated from marine environments on four potential carbon sources: glucose, xylose, galactose and glycerol. Growth was assessed without addition of additives to avoid potential competing carbon sources, even if poorly utilised, and to minimise the preparation time. The aim was to determine whether a simple procedure, with no optimisation for individual strains (*cf*. [68]) would provide consistent kinetic growth data for strain assessment that could be used to guide process scale-up for strains of interest. The fungi characterised in this study were isolated from marine environments, since there are now numerous compounds of interest identified from marine isolates [74–77], but little information on their growth kinetics. The methods are also applicable to fungi isolated from other environments.

## Materials and methods

### Strains

*Penicillium pinophilum* LF458, *Microascus brevicaulis* (formerly *Scopulariopsis brevicaulis* [78]) LF580, *Tritiracium* sp. LF562, *Calcarisporium* sp. KF525, *Penicillium chrysogenum* KF657 and KF654, *Dendryphiella salina* LF304, *Asteromyces cruciatus* LF680 and *Halenospora varia* KF560 were obtained from the culture collection of the Kiel Center for marine natural products at GEOMAR, Helmholtz Centre for Ocean Research Kiel, as a kind gift from A. Labes and J. F. Imhoff. Stock cultures were maintained as mycelia on agar-solidified medium (potato dextrose agar containing 25 g L^-1^ Tropic Marin® sea salt) at 6°C, as conidia or mycelial fragments suspended in 20% v/v glycerol, 0.8% w/v NaCl with ~0.025% v/v Tween 20 at -80°C or on Microbank™ Bacterial and Fungal Preservation System beads (Pro-Lab Diagnostics, UK) at -80°C.

### Inoculum preparation

Conidia were collected from sporulating (LF304, KF657, KF525, LF562, LF458 and LF580) cultures on agar-solidified medium in 5 mL of a solution containing 200 g L^-1^ glycerol, 8 g L^-1^ NaCl and 0.25 g L^-1^ Tween® 20 and stored at -80°C, if not used fresh.

Mycelial suspensions (*H*. *varia* KF560 and *A*. *cruciatus* LF680) were prepared by transferring small pieces of fungal mycelium (4 or 5 pieces, 4–9 mm^2^), excised from mycelia growing on agar-solidified medium, to a 2 mL screw cap microcentrifuge tube containing 600 μl NaCl (9 g L^-1^) and approximately 0.7 g zirconium beads (1 mm diameter). The mycelia were homogenized in a Precellys®24 homogenizer (Bertin Technologies, France) for 5 seconds at 5000 rpm. The mycelial homogenate of *A*. *cruciatus* LF680 was diluted 15-fold in sterile water and used fresh. To assess a process for storing mycelia at -80°C for later use, fresh mycelial homogenate of *H*. *varia* KF560 was inoculated to 10 mL yeast extract (10 g L^-1^) peptone (20 g L^-1^) dextrose (20 g L^-1^) medium containing 4 g L^-1^ agar and 28 g L^-1^ Tropic Marin® sea salt in a 50 mL flask, and incubated at 24°C until dispersed, filamentous growth was observed. Sterile glycerol was added to the culture to a final concentration of 200 g L^-1^ and the suspension stored at -80°C without washing. Inoculum for microtiter plates was prepared from frozen stock by diluting the mycelial suspension 30-fold in sterile water.

### Media

The medium for growth in microtiter plates was adapted from that described by Verduyn et al. [79], adding 28 g L^-1^ Tropic Marin® sea salt and 33 g L^-1^ PIPPS buffer, with the pH adjusted to 4.25. The media contained 5.0 g L^-1^ (NH_4_)_2_SO_4_, 3.0 g L^-1^ KH_2_PO_4_, 0.5 g L^-1^ MgSO_4_·7H_2_O, 15 mg L^-1^ EDTA, 4.5 mg L^-1^ ZnSO_4_·7H_2_O, 1.0 mg L^-1^ MnCl_2_·2H_2_O, 0.3 mg L^-1^ CoCl_2_·6H_2_O, 0.3 mg L^-1^ CuSO_4_·5H_2_O, 0.4 mg L^-1^ Na_2_MoO_4_·2H_2_O, 4.5 mg L^-1^ CaCl_2_·2H_2_O, 3.0 mg L^-1^ FeSO_4_·7H_2_O, 1.0 mg L^-1^ H_3_BO_4_, 0.1 mg L^-1^ KI, 0.05 mg L^-1^ D-biotin, 1.0 mg L^-1^ CaPantothenate, 5.0 mg L^-1^ nicotinic acid, 25 mg L^-1^ myo-inositol, 1.0 mg L^-1^ thiamine HCl, 1.0 mg L^-1^ pyridoxine HCl, and 0.2 mg L^-1^ p-aminobenzoic acid. The amount of carbohydrate (glucose, xylose, galactose or glycerol) was reduced to 2 g L^-1^ to limit biomass production, so that maximum OD measurements would reflect substrate use. Some wells contained medium to which no carbon source was added, to serve as control for carbon added as glycerol with the inoculum.

### Cultivation conditions

BioScreen (Bioscreen C MBR automated turbidometric analyser, Growth Curves Ltd, Finland) cultivations were carried out in 100-Well Honeycomb 2 plates (Oy Growth Curves Ab Ltd., well depth 14 mm, well diameter 7 mm at top) containing 290 μl medium and 10 μl inoculum, providing final conidium concentrations of 1 to 6 x 10^4^ conidia per mL for sporulating strains. An exception was *P*. *chrysogenum* KF657, which was inoculated with 4 x 10^5^ conidia per mL. The mycelial homogenate of *A*. *cruciatus* LF680 was diluted 15-fold in water and 10 μl used as inoculum. *H*. *varia* KF560 was inoculated as frozen mycelial suspension. Dilution of the frozen suspension 30 fold prior to inoculation reduced the transferred glycerol to 0.22 g L^-1^, with glucose and peptone from the inoculum contributing less than 22 mg L^-1^ to the well cultivation. 100-well plates were incubated at 24°C with continuous shaking at medium speed and maximum amplitude (at a frequency of 10 Hz, providing 600 rpm in a linear, rather than orbital motion). Optical density (OD) was measured at 600 nm at 30 min intervals for up to 10 days.

For growth in 96-well plates, Nunc™ Flat Bottom 96-well Clear Polystyrene Plates (Thermo Scientific 260860; well depth 11.4 mm, well diameter 7 mm at top) containing 145 μl medium were inoculated with 7.5 μl conidial or mycelial suspension and incubated at 24°C with 1100 rpm agitation in a Thermo Scientific Cytomat plate hotel (throw = 1 mm; holding up to 23 plates). Dilution of conidia provided final concentrations of ~5 x 10^4^ mL^-1^. Mycelial homogenate of LF680 was diluted 22 fold and the frozen mycelia of KF560 were diluted 45 fold. Growth was measured as increase in OD at 595 nm at 2 or 3 h intervals, using a DTX 880 multimode detector (Beckman Coulter) associated with the Cytomat plate hotel. OD values for *P*. *chrysogenum* KF657, *D*. *salina* LF304, *H*. *Varia* KF560 and *A*. *cruciatus* LF680 were obtained without removal of the lid from the plates to prevent risks of contamination. OD values for *P*. *pinophilum* LF458, *M*. *brevicaulis* LF580, *Tritiracium* sp. LF562 and *Calcarisporium* sp. KF525 were measured with lid removal so that condensation on the lids did not distort the OD values. In some cultures, water was added to the outer wells of 96-well plates, to compensate for evaporation of liquid from wells, particularly at the edges of plates in the Cytomat plate hotel.

**Fig 1:**
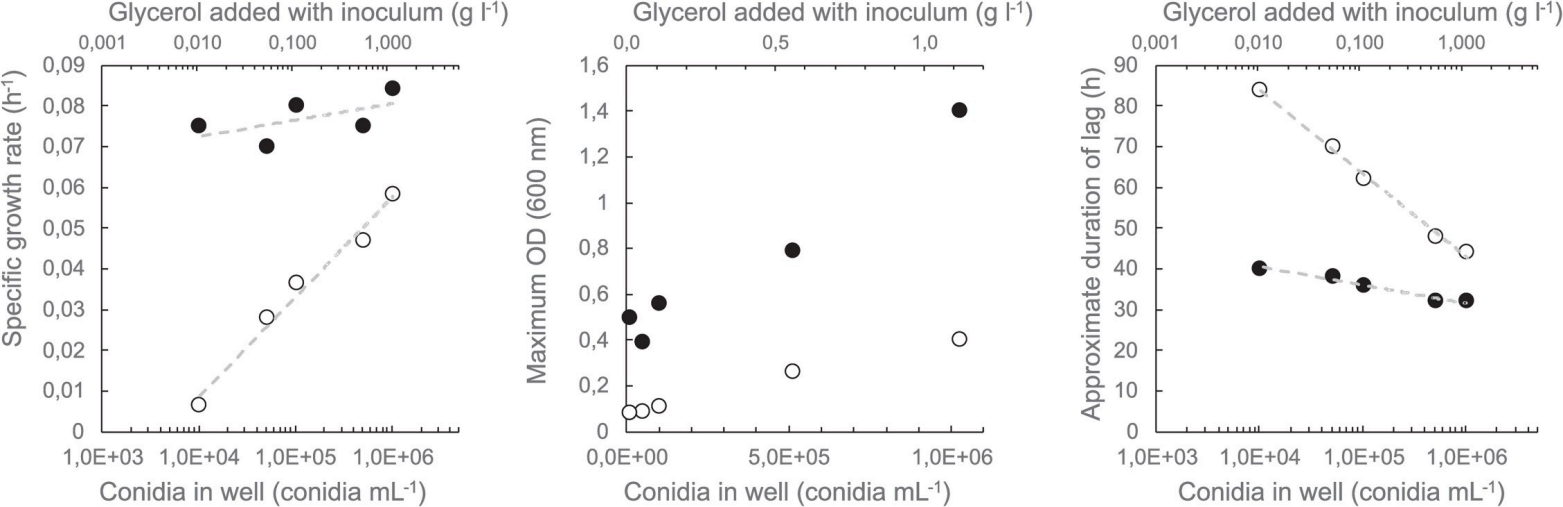
Effect of glycerol and conidium concentration in the inoculum on measurement of specific growth rate, maximum OD and approximate duration of lag phase for *P*. *chrysogenum* LF654. Mycelia were grown in defined medium buffered at pH 4.25 with (solid symbols) or without (open symbols) added glucose (2 g L^-1^) in 300 μL cultures in 100-well plates in a BioScreen C.

**Fig 2:**
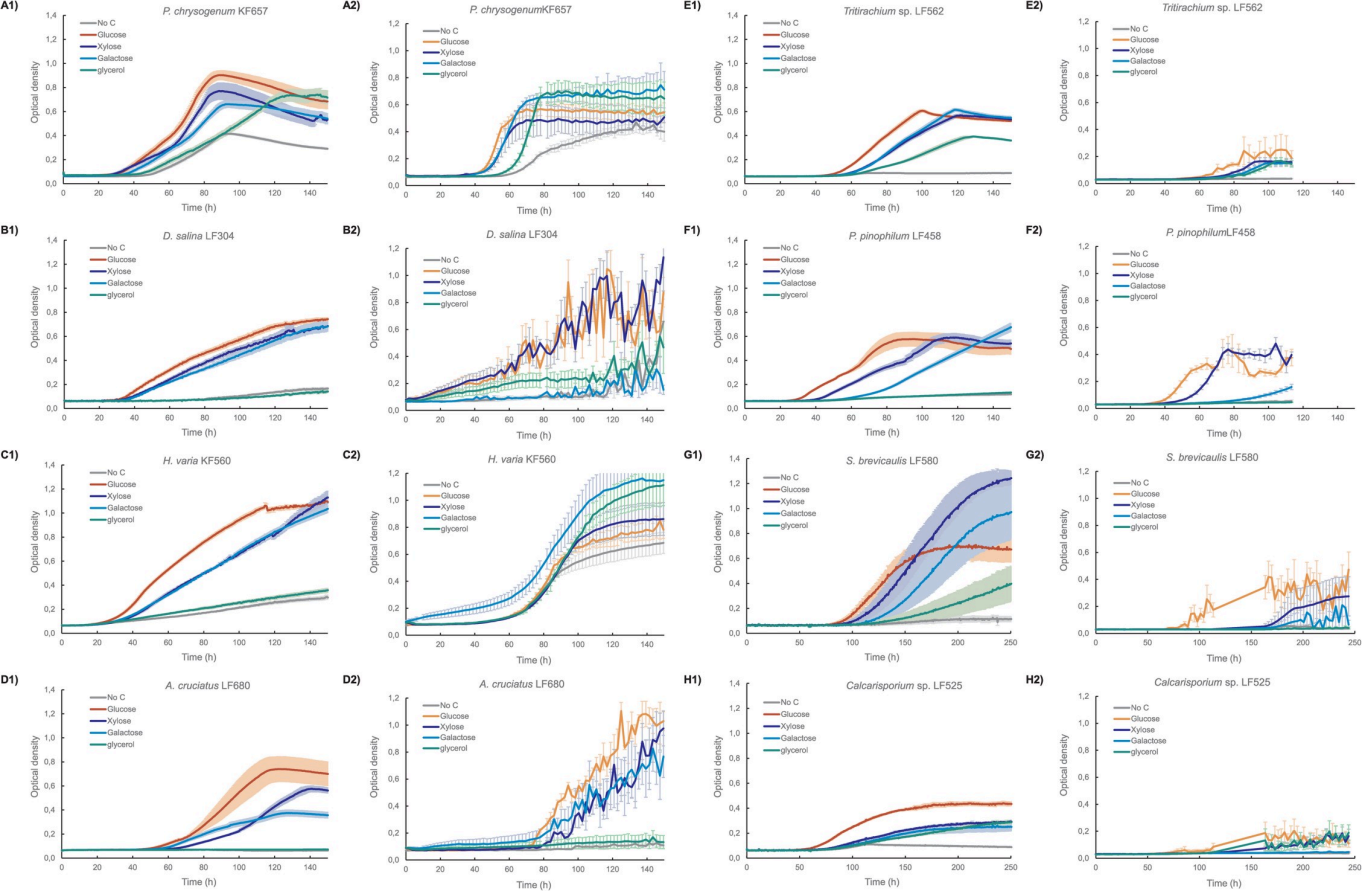
Optical density of A) *P*. *chrysogenum* KF657, B) *D*. *salina* LF304, C) *H*. *varia* KF560, D) *A*. *cruciatus* LF680, E) *Tritiracium* sp. LF562, F) *P*. *pinophilum* LF458, G) *M*. *brevicaulis* LF580 and H) *Calcarisporium* sp. KF525. Mycelia were grown in defined medium buffered at pH 4.25 with glucose (red), xylose (blue), galactose (cyan) or glycerol (green) as carbon source, or no added carbon source (grey) in 300 μl cultures in 100-well plates in a BioScreen C (1) or 150 μl cultures in 96-well plates in a Cytomat plate hotel (2). Error bars represent ± standard error of the mean (sem) for 3 to 6 replicates. Note that a short break in the power supply disturbed data collection after 110 hours for *M*. *breviaculis* LF580 and *Calcarisporium* sp. KF525. Data collection was restored after 160 hours.

Photographs of the 100-well Honeycomb 2 and 96-well plates used in these cultivations are included in the stored data MicrotiterPlate_photographs file, Data Figs 1 & 2 [80].

### Determination of maximum specific growth rate and maximum OD

Specific growth rates and maximum OD were determined by the on-line Specific Growth Rate Calculation program by S. Castillo and D. Barth (https://scsandravtt.shinyapps.io/SGRapp/), based on the Grofit package for R [81]. This program is designed for calculation of specific growth rates from BioScreen output, but can be used with any data in csv format, with time in the first column and one row of headings. In order to improve the calculation of specific growth rate from noisy data (e.g. caused by random spikes that result from movement of pellets in and out of the measurement zone), cubic smoothing splines are fit to the input data. The degree of smoothing is specified by the user through the smoothing factor, which typically ranges from 0 to 1. Smoothing reduces the noise in the data, eliminating the need for manual, arbitrary curation. Both the original data and the smoothed curves can be visualised in the program. The program determines the maximum specific growth rate from the entire smoothed data set. Subjective determination of the exponential phase is not necessary. A high smoothing factor (0.7) was used for data from 96-well plates with poor or no growth, in order to eliminate the effect of random spikes in the data, whereas a low smoothing factor (0.3) was used for data from cultures with good growth. The smoothing factor had less impact on the results from 100-well plates and a smoothing factor of 0.7 was used for all.

### Statistics

Data are presented as mean ± standard error of the mean. Analysis of variance was used to compare specific growth rates or maximum OD values for the same strain growing on different carbon sources, with significant differences identified using Fisher's multiple range test. The student-t test was used to compare specific growth rates obtained in the Bioscreen C (100-well plates) with those obtained for the same strain and condition in the Cytomat plate hotel (96-well plates).

## Results and discussion

### Effect of inoculum and glycerol concentration on growth

Conidial and mycelial suspensions were stored in glycerol at -80°C. Some strains were able to grow on glycerol as a carbon source. *P*. *chrysogenum* LF654 was used to assess the impact of glycerol in the inoculum, along with the inoculum size, on subsequent growth in the BioScreen C. Growth was assessed in the presence and absence of added glucose [80]. *P*. *chrysogenum* LF654 grew on glycerol, although not as well as on glucose (Fig 1, [80]). The glycerol concentration did not affect the specific growth rate when glucose was present in the medium (Fig 1), but allowed growth in its absence, indicating that glycerol in the inoculum could affect the interpretation of how well poor carbon sources were consumed. The addition of glycerol with the inoculum did affect the maximum OD observed when glucose was present as the main carbon source (Fig 1).

The inoculum concentration for five of the six sporulating strains included in the comparison of growth in the BioScreen C with that in the Cytomat plate hotel was reduced from 5 x 10^5^, as recommended by [68] and [69], to 1–6 x 10^4^ conidia mL^-1^. However, cultures with *P*. *chrysogenum* KF657 had already been completed using 4 x 10^5^ conidia mL^-1^ and it was clear that glycerol contributed to growth in the medium with no added carbon (Fig 2). Similarly, cultures of *H*. *varia* KF560, which were inoculated with mycelial suspension frozen in glycerol also contained sufficient glycerol to allow growth in medium to which carbohydrate had not been added (Fig 2).

### Characteristics of mycelial growth in the Bioscreen C and Cytomat plate hotel

**Fig 3:**
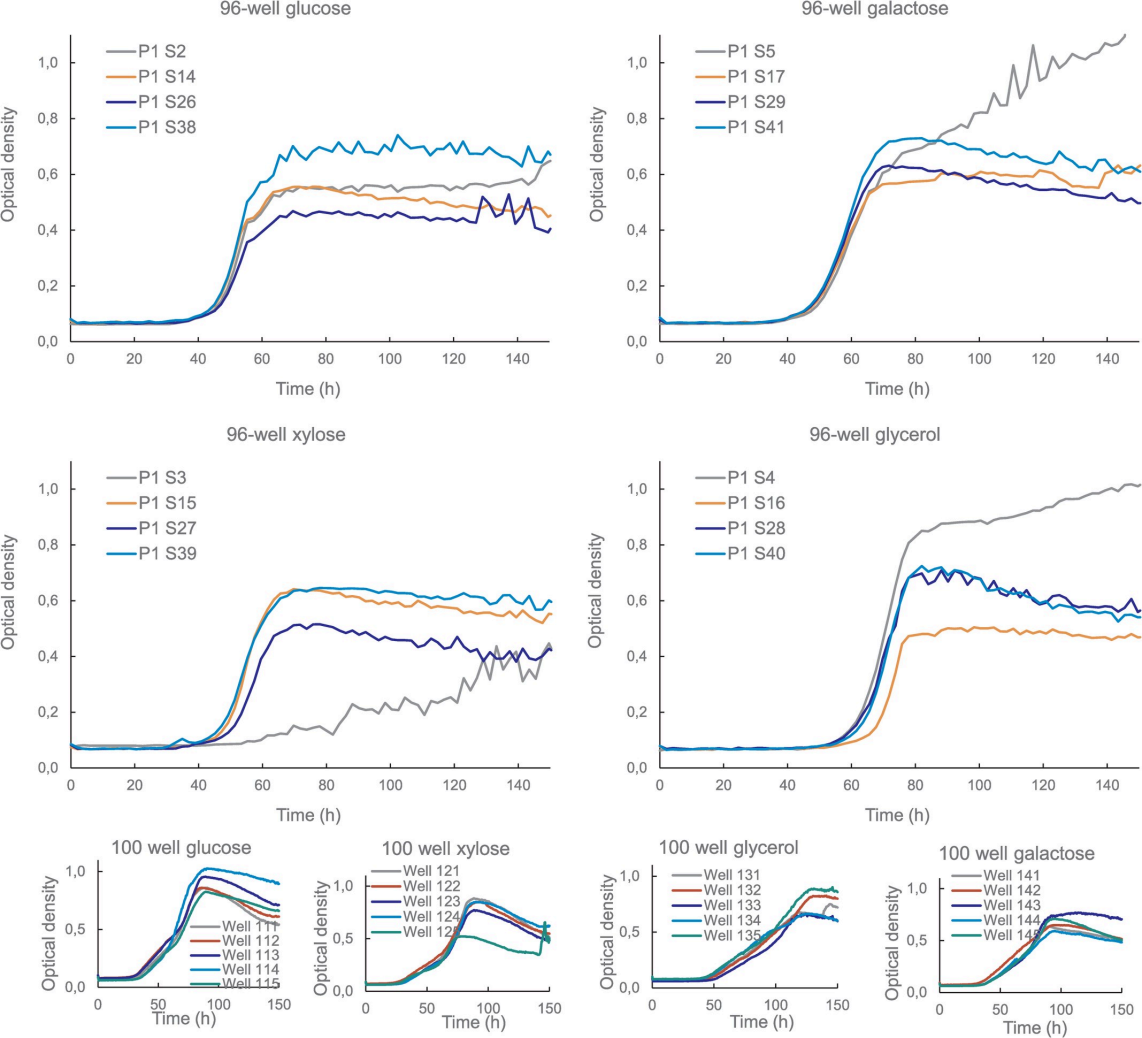
Optical density of *P*. *chrysogenum KF657* grown in defined medium buffered at pH 4.25 with glucose, xylose, galactose or glycerol as carbon source, in 150 μl cultures in 96-well plates in a Cytomat plate hotel (large graphs) or in 300 μl cultures in 100-well plates in a BioScreen C (small graphs). Edge wells (grey) in the 96-well plates are indicated as P1 S2, P1 S3, P1 S5 and P1 S4 and in the 100-well plates as Well 111, Well 121, Well 141 and Well 131.

All fungi tested grew in both the Bioscreen C (100-well plates, with moderate shaking) and Cytomat plate hotel (96-well plates with 1100 rpm) (Fig 2, [80]). Mycelia were more likely to aggregate into pellets when grown in the Cytomat plate hotel than in the Bioscreen C, whereas mycelia sometimes attached to the walls of the wells in the Bioscreen C, leaving a clear area in the centre of the well (see Data Fig 3, MicrotiterPlate_photographs [80]). The presence of one or more pellets resulted in noise in the OD measurements and variation between replicate wells. The variation between replicates is seen in the large error bars (relative standard error of 10–30%, sometimes higher) of the Cytomat cultures (Fig 2). In contrast, growth in the Bioscreen C plates was mostly dispersed, with good reproducibility between wells (relative standard error of 4–10%, except LF580; Fig 2). Addition of Tween 80 [56] or agar [68] to the medium may have reduced attachment and pellet formation, but was not used in order to keep the assay as simple as possible and to avoid additives that could potentially serve as carbon sources.

In addition to problems with pellet formation in the wells, the Cytomat plate hotel cultures suffered from condensation on the lids of the 96-well plates (see Data Fig 4, MicrotiterPlate_photographs [80]) and evaporation of liquid from wells at the edges of plates. Condensation on the lids contributed to the measured OD value when lids were not removed for measurements. When lids were removed, maximum OD values were much lower (Table 1, *Tritirachium* sp., *P*. *pinophilum*, *M*. *brevicaulis*, *Calcarisporium* sp.) than when they were not (Table 1, *P*. *chrysognenum*, *D*. *salina*, *H*. *varia*, *A*. *cruciatus*). Evaporation in edge wells of 96-well plates resulted in high OD values, particularly during stationary phase (as observed in parallel wells of *P*. *chrysogenum* KF657 on galactose and glycerol, Fig 3). However, evaporation effects were not equal in all edge wells, as seen in the *P*. *chrysogenum* KF657 examples in Fig 3: there was almost no evaporation from well 2 (glucose) and poor growth in well 3 (xylose). Evaporation was not expected to affect the maximum specific growth rate of strains which reached stationary phase within less than 80 h, but the effect was avoided in cultures of LF562, LF580, KF525 and LF458 by filling outer wells with water [82]. This reduced the number of replicates or conditions which were measured in a single plate. Since the Cytomat plate hotel holds up to 23 plates, the reduction in usable wells per plate would be less restrictive in terms of screening than being limited to 200 wells in one BioScreen C growth test.

**Table 1:** Maximum OD values for marine fungi cultivated in microtiter plates.

	System	Glucose	Xylose	Galactose	Glycerol	No C
*P*. *chrysogenum* KF657	BSC	0.90 ± 0.04 ^c^	0.78 ± 0.06 ^b^	0.67 ± 0.03 ^b^	0.75 ± 0.05 ^b^	0.42 ± 0.01 ^a^
	Cph	0.57 ± 0.05^ABC^	0.60 ± 0.05^AB^	0.74 ± 0.09 ^C^	0.71 ± 0.09 ^BC^	0.40 ± 0.03 ^A^
*D*. *salina* LF304	BSC	0.74 ± 0.01 ^b^	0.69 ± 0.03 ^b^	0.69 ± 0.04 ^b^	0.14 ± 0.01 ^a^	0.17 ± 0.01 ^a^
	Cph	0.99 ± 0.15 ^C^	0.91 ± 0.06 ^C^	0.20 ± 0.05 ^AB^	0.32 ± 0.05 ^B^	0.18 ± 0.06 ^A^
*H*. *varia* KF560	BSC	1.09 ± 0.01 ^bc^	1.13 ± 0.05 ^c^	1.04 ± 0.03 ^b^	0.36 ± 0.02 ^a^	0.30 ± 0.02 ^a^
	Cph	0.80 ± 0.07 ^A^	0.87 ± 0.12 ^A^	1.18 ± 0.20 ^A^	1.14 ± 0.17 ^A^	0.72 ± 0.08 ^A^
*A*. *cruciatus* LF680	BSC	0.74 ± 0.10^d^	0.58 ± 0.02 ^c^	0.38 ± 0.03 ^b^	0.07 ± 0.00 ^a^	0.07 ± 0.00 ^a^
	Cph	1.08 ± 0.05 ^B^	1.08 ± 0.12 ^B^	0.99 ± 0.18 ^B^	0.14 ± 0.05 ^A^	0.13 ± 0.03 ^A^
*Tritirachium* sp. LF562	BSC	0.59 ± 0.01 ^cd^	0.57 ± 0.01 ^c^	0.60 ± 0.01 ^d^	0.39 ± 0.01 ^b^	0.09 ± 0.00 ^a^
	Cph	0.14 ± 0.02 ^B^	0.17 ± 0.03 ^B^	0.15 ± 0.02 ^B^	0.18 ± 0.03 ^B^	0.04 ± 0.00 ^A^
*P*. *pinophilum* LF458	BSC	0.58 ± 0.06 ^b^	0.59 ± 0.03 ^b^	0.68 ± 0.03 ^b^	0.13 ± 0.00 ^a^	0.12 ± 0.00 ^a^
	Cph	0.59 ± 0.08 ^B^	0.52 ± 0.02 ^B^	0.15 ± 0.02 ^A^	0.05 ± 0.00 ^A^	0.05 ± 0.01 ^A^
*M*. *brevicaulis* LF580	BSC	0.71 ± 0.10 ^bc^	1.26 ± 0.05 ^d^	0.98 ± 0.21 ^cd^	0.41 ± 0.14 ^ab^	0.12 ± 0.02 ^a^
	Cph	0.39 ± 0.06 ^B^	0.28 ± 0.15 ^AB^	0.14 ± 0.11 ^AB^	0.04 ± 0.01 ^A^	0.05 ± 0.01 ^A^
*Calcarisporium* sp. KF525	BSC	0.44 ± 0.02 ^c^	0.30 ± 0.01 ^b^	0.26 ± 0.04 ^b^	0.29 ± 0.02 ^b^	0.11 ± 0.00 ^a^
	Cph	0.17 ± 0.06 ^AB^	0.17 ± 0.02 ^AB^	0.05 ± 0.00 ^AB^	0.18 ± 0.06 ^b^	0.04 ± 0.00 ^A^

Marine fungi were grown in 100-well plates incubated in a BioScreen C (BSC) or 96-well plates incubated in a Cytomat plate hotel (Cph) with 2 g L^-1^ glucose, xylose, galactose or glycerol as substrate. No C refers to wells containing medium to which no carbohydrate was added. Data are the average of 3 to 6 replicates from the Cytomat hotel and 5 replicates from the BioScreen cultures, ± sem. Values in the same row with the same superscript (a to e for BioScreen or A to D for Cytomat) did not differ significantly (p > 0.05, Fisher's multiple range test).

Specific growth rates were determined for cultures incubated in both the Cytomat plate hotel and the BioScreen C (Table 2), even though some strains grew poorly in the Cytomat plate hotel. Using a program to automatically apply a smoothing factor and determine the specific growth rate removed subjective judgement from the calculation. Similar specific growth rates (p > 0.05) were observed for most cultures growing in the BioScreen C as in the Cytomat plate hotel (Table 2), with some exceptions. Higher (p < 0.05) specific growth rates were observed in the Cytomat for *P*. *chrysogenum* KF657 and *P*. *pinophilum* LF458 than in the BioScreen C on all substrates, and for *A*. *cruciatus* LF680, *Tritirachium* LF562, *H*. *varia* KF560 and *D*. *salina* LF304 growing on xylose (Table 2). *D*. *salina* also had high specific growth rates on glucose and glycerol, but because the replicates in the Cytomat plate hotel differed considerably from each other these did not differ statistically from those in the BioScreen C. On the other hand, *D*. *salina* LF304 grew on galactose and *M*. *breviaulis* LF580 on glycerol in the BioScreen C, but not in the Cytomat (Fig 2). Otherwise general conclusions about growth (ability to grow and preference for substrates) were similar regardless of the system used.

**Table 2:** Specific growth rate (h^-1^) of marine fungi cultivated in microtiter plates.

Strain	System	Glucose	Xylose	Galactose	Glycerol	No C
*P*. *chrysogenum* KF657	BSC	0.07 ± 0.00^d^	0.06 ± 0.01^bc^	0.06 ± 0.00^c^	0.05 ± 0.00^A^	0.05 ± 0.00^ab^
	Cph	0.17 ± 0.01^D^*	0.15 ± 0.01^C^*	0.12 ± 0.00^B^*	0.15 ±0.01^CD^*	0.06 ± 0.00^A^*
*D*. *salina* LF304	BSC	0.07 ± 0.00^d^	0.05 ± 0.00^c^	0.05 ± 0.00^c^	0.01 ± 0.00^a^	0.02 ± 0.00^bc^
	Cph	0.27 ± 0.06^B^	0.20 ± 0.04^B^*	0.03 ± 0.01^A^	0.11 ± 0.03^B^	0.03 ± 0.01^A^
*H*. *varia* KF560	BSC	0.07 ± 0.00^e^	0.05 ± 0.00^d^	0.05 ± 0.00^c^	0.02 ± 0.00^b^	0.02 ± 0.00^a^
	Cph	0.07 ± 0.01^AB^	0.06 ± 0.00^A^*	0.05 ± 0.00^A^	0.09 ± 0.02^B^*	0.05 ± 0.00^A^
*A*. *cruciatus* LF680	BSC	0.05 ± 0.01^c^	0.04 ± 0.00^b^	0.04 ± 0.00^bc^	0.00 ± 0.00^a^	0.01 ± 0.00^a^
	Cph	0.07 ± 0.00^C^	0.05 ± 0.01^B^*	0.04 ± 0.01^B^	0.01 ± 0.00^A^	0.01 ± 0.01^A^
*Tritirachium* sp. LF562	BSC	0.07 ± 0.00^e^	0.05 ± 0.00^c^	0.06 ± 0.00^d^	0.04 ± 0.00^b^	0.02 ± 0.00^a^
	Cph	0.07 ± 0.01^B^	0.07 ± 0.00^B^*	0.07 ± 0.01^B^	0.09 ± 0.02^B^*	0.01 ± 0.00^A^
*P*. *pinophilum* LF458	BSC	0.07 ± 0.00^d^	0.06 ± 0.00^c^	0.04 ± 0.00^b^	0.01 ± 0.00^a^	0.01 ± 0.00^a^
	Cph	0.17 ± 0.01^D^*	0.13 ± 0.01^C^*	0.03 ± 0.00^B^*	0.01 ± 0.00^A^*	0.01 ± 0.00^A^
*M*. *brevicaulis* LF580	BSC	0.05 ± 0.01^c^	0.05 ± 0.00^c^	0.04 ± 0.00^bc^	0.02 ± 0.00^ab^	0.01 ± 0.00^a^
	Cph	0.06 ± 0.01^B^	0.06 ± 0.01^B^	0.02 ± 0.01^A^	0.01 ± 0.00^A^	0.01 ± 0.01^A^
*Calcarisporium* sp. KF525	BSC	0.04 ± 0.00^c^	0.02 ± 0.00^b^	0.02 ± 0.00^a^	0.02 ± 0.00^a^	0.02 ± 0.01^a^
	Cph	0.02 ± 0.01^B^	0.03 ± 0.01^B^	0.01 ± 0.00^A^*	0.025 ±0.01^B^	0.01 ± 0.00^A^

Marine fungi were grown in 100-well plates incubated in a BioScreen C (BSC) or 96-well plates incubated in a Cytomat plate hotel (Cph) with glucose, xylose, galactose or glycerol as substrate. No C refers to wells containing medium to which no carbohydrate was added. Data are the average of 3 to 6 replicates from the Cytomat hotel and 5 replicates from the BioScreen, ± sem. Values in the same row with the same superscript (a to e, BioScreen; or A to D, Cytomat hotel) did not differ significantly (p > 0.05, Fisher's multiple range test).

* indicates that the measurement in the BioScreen C was significantly (p < 0.05) different from that in the Cytomat plate hotel.

### Growth of marine fungi on glucose, xylose, galactose and glycerol

All eight marine fungi tested grew on glucose, xylose and galactose, whereas only *P*. *chrysogenum* KF657, *H*. *varia* KF560, *Tritiracium* sp. LF562, *M*. *brevicaulis* LF580, and *Calcarisporium* sp. KF525 grew on glycerol (Table 2, Fig 2). Medium lacking a carbon source was used as a control for background growth, i.e. growth on glycerol (or yeast extract and peptone) which was added with the inoculum. The specific growth rate was always significantly lower (p < 0.05) when no carbon was added to the medium than on glucose (Table 2). Strains (KF657 and KF560) which received too much glycerol with the inoculum had measureable specific growth rates with no other added carbon, but produced less biomass (lower OD; Table 1) than with 2 g L^-1^ glycerol. Based on the measurements in the BioScreen, specific growth rates were highest (p < 0.05) on glucose (μ = 0.04 to 0.07 h^-1^) for all strains except *M*. *brevicaulis* LF580. *M*. *brevicaulis* LF580 grew equally well on glucose, xylose and galactose (Table 2) and produced high ODs on all three substrates (Table 1). Most strains preferred xylose to galactose and specific growth rates on glycerol were lower than on either, if glycerol was consumed (Table 2).

Specific growth rates for two of the strains, *M*. *brevicaulis* LF580 and *Calcarisporium* sp. KF525, have previously been reported. The specific growth rates measured in microtiter plates of the slow growing KF525 (0.02 to 0.04 h^-1^, Table 2) were comparable to that previously published for growth in defined medium on glucose (0.03 h^-1^, [83]). However, *M*. *brevicaulis* LF580 apparently grew much faster in pH controlled, fully aerated bioreactors (0.17 h^-1^ on glucose and 0.14 h^-1^ on xylose, [20]) than in the microtiter plates (0.05 h^-1^ in the BioScreen C and 0.06 h^-1^ in the Cytomat hotel), suggesting that fast growing fungi may not show their full potential in the microtiter plates. It is probable that fast-growing filamentous fungi suffer oxygen limitation in the microtiter plates because of the viscous nature of the hyphae and the limitation would result in low measurements of the maximum specific growth rate. Strains which grow as pellets would be less viscous, but still oxygen limited within the pellets. Slow growing fungi, such as *Calcarisporium* sp. KF525 have lower oxygen demand because of their slow growth and thus the specific growth rate is closer to that measured in optimal conditions than for the fast growing strain.

The microtiter plate thus provides valuable insight into substrate preference and some perspective on the kinetics of growth, but not necessarily for optimal growth. Kinetic data are still needed from more optimal conditions. None-the-less, as a method for preliminary characterisation of strains for which other data is not available, the microtiter plate provides a way to obtain kinetic data in a range of conditions for multiple strains within a short time. However, for a limited number of strains it could be useful to include a second characterisation step, in which more optimal conditions would be used, e.g. by including a dispersal agent such as Tween 80 or agar to reduce attachment to walls and promote filamentous growth [56, 68].

Growth was quantified in terms of maximum OD values (Fig 2, Table 1), as well as maximum specific growth rates. We note, however, that because of differences in the strain morphology (filamentous growth, branch frequency, tendency to make pellets), OD values between strains may not compare directly [23]. Nor was the correlation in OD values between the BioScreen C reader and the DTX 880 multimode detector used in combination with the Cytomat plate hotel determined, so maximum OD values should not be compared between BioScreen C and Cytomat plate hotel cultures.

Long lag phases (30–100 h) were observed for all strains in all conditions, and were generally longer for cultures in the Cytomat plate hotel than in the BioScreen C (Fig 2). The lag phase was affected by the inoculum concentration (Fig 1), which was kept low to minimise growth on glycerol in strains which were able to utilise it. The lag phase could be reduced by using a larger inoculum, but conidia (or mycelia) should then first be washed to remove glycerol, which would increase the preparation time with the microtiter plates, reducing the throughput.

## Conclusion

Eight marine fungi were characterised for their ability to grow on four substrates in either a BioScreen C or a Cytomat plate hotel. Strains grew better and replicates were more similar in the BioScreen C than in the Cytomat (Fig 2). The high variability between replicates in the Cytomat plate hotel meant that it was generally only possible to distinguish (statistically) between growth and no growth, but not to distinguish whether or not the strain grew better on xylose, glucose or galactose (Tables 1 and 2). Since the replicates showed less variation in the BioScreen C than in the Cytomat hotel, it could be seen that most strains preferred glucose to xylose and xylose to galactose (Tables 1 and 2).

Differences in humidity control and shaking probably contributed to the growth differences in the BioScreen and Cytomat plate hotel. Development of optimal mixing parameters for the plate hotel, with water in the edge wells to reduce evaporation, would be useful to obtain higher screening throughput than is possible in the BioScreen, with its limit of two plates per screen. However, both systems can provide physiological data with filamentous fungi, including strains which produce pellets, as long as sufficient replicates are included, even without extensive inoculum optimisation. The kinetic data may not reflect optimal growth of some strains, but provides a starting point for further characterisation.
